# Supplementary material for: Geographical Distribution of Dietary Patterns and Their Association with T2DM in Chinese Adults Aged 45 y and Above: A Nationwide Cross-Sectional Study
Source: Nutrients. 2023 Dec 28;16(1):107. doi: 10.3390/nu16010107 (PMC10780680; doi:10.3390/nu16010107)
Supplement: Supplementary file 1 [file nutrients-16-00107-s001.zip › Supplementary Tables.pdf]

Table S1 Food grouping used in dietary pattern analysis

Food Groups	Food Groups
Rice and Products	rice and products
Wheat and Products	wheat and products
Other Cereals	corn and products, buckwheat, millet
Fungi and Algae	mushrooms, black fungus, white fungus, kelp, seaweed, nori
Tubers	potatoes, taro, sweet potatoes
Dry Legume	mung beans, red beans, kidney beans
Legume Products	soybeans, soy milk, tofu pudding, Tofu, bean curd sheets, shredded tofu
Vegetables	vegetables
Fruits	fruits
Nuts	sunflower seeds, peanuts, walnuts, etc
Pork	pork
Other Red Meats	beef, lamb, donkey meat, etc
Meat Products and Offal	meat products, animal offal
Poultry	poultry
Aquatic Products	fish, shrimp, crab, mollusks, etc
Eggs	fresh eggs, salted eggs, century eggs
Milk and Dairy Products	milk, milk powder, yogurt, cheese
Liquors	Chinese spirits, beer, rice wine, grape wine
Beverages	carbonated beverages, fruit and vegetable juice drinks, coffee, etc
Snacks	snacks

Table S2 Characteristics of subjects according to Dietary Pattern scores quartile distribution

	Q1<br>N=9162	Q2<br>N=9162	Q3<br>N=9162	Q4<br>N=9162	P-value	P-trend
**Oriental traditional Pattern**						
**Female (n, %)**	5113(55·8)	5149(56·2)	4955(54·1)	4765(52·0)	<0·001	<0·001
**Age (Median,Q1, Q3,y)**	59·2(51·8;65·9)	58·4(51·3;64·8)	58·3(51·5;64·9)	58·3(51·4;64·6)	<0·001	<0·001
**Age group (n, %)**					<0·001	<0·001
**45-59 y**	4856(53·0)	5120(55·9)	5165(56·4)	5156(56·2)		
**60-74 y**	3714(40·5)	3620(39·5)	3624(39·6)	3644(39·8)		
**≥75 y**	592(6·5)	422(4·6)	373(4·0)	362(4·0)		
**Rural (n, %)**	6636(72·4)	5839(63·7)	5117(55·9)	4396(48·0)	<0·001	<0·001
**Income (n, %)**					<0·001	<0·001
**<5000 Yuan/month**	7792(85·1)	7396(80·7)	7142(78·0)	6651(72·6)		
**5000-9999 Yuan/month**	1037(11·3)	1376(15·0)	1553(17·0)	1973(21·5)		
**≥10000 Yuan/month**	333(3·6)	390(4·3)	467(5·0)	538(5·9)		
**Han ethnicity (n, %)**	7781(84·9)	8290(90·5)	8514(92·9)	8706(95·0)	<0·001	<0·001
**Educational level (n, %)**					<0·001	<0·001
**Below junior high school**	6507(71·0)	5746(62·7)	4798(52·4)	3746(40·9)		
**Junior high school**	2531(27·6)	3231(35·3)	3986(43·5)	4778(52·1)		
**Senior high school or above**	124(1·4)	185(2·0)	378(4·1)	638(7.0)		
**Having a partner (n, %)**	8524(93·0)	8641(94·3)	8715(95·1)	8739(95·4)	<0·001	<0·001
**Adequate physical activity (n, %)**	8326(90·9)	8254(90·1)	8073(88·1)	8090(88·3)	<0·001	<0·001
**Smoking (n, %)**	2567(28·0)	2418(26·4)	2416(26·4)	2310(25·2)	<0·001	<0·001
**Drinking (n, %)**	3021(33·0)	3127(34·1)	3311(36·1)	3672(40·1)	<0·001	<0·001
**BMI(Median, P25th, P75th,kg/m^2)**	23·2(21·1;25·6)	23·9(21·7;26·3)	24·5(22·2;26·7)	24·8(22·6;27·1)	<0·001	<0·001
**BMI group (n, %)**					<0·001	<0·001
**Underweight**	459(5·0)	333(3·6)	206(2·3)	166(1·8)		
**Normal weight**	4928(53·8)	4337(47·3)	3860(42·1)	3561(38·9)		
**Overweight**	2899(31·6)	3249(35·5)	3687(40·2)	3753(41·0)		
**Obesity**	876(9·6)	1243(13·6)	1409(15·4)	1682(18·3)		
**Family history of chronic diseases (n, %)**	3029(33·1)	3934(42·9)	4586(50·1)	5154(56·3)	<0·001	<0·001
**Diabetes Mellitus (n, %)**	819(8·9)	922(10·1)	1096(12·0)	1146(12·5)	<0·001	<0·001
**FPG(Median, P25th, P75th,mmol/L)**	5·2(4·8;5·7)	5·3(4·9;5·8)	5·3(4·9;5·8)	5·3(4·9;5·8)	<0·001	<0·001
**HbA1c (mean, std)**	5·1(0·9)	5·1(1·0)	5·2(1·0)	5·2(1·0)	<0·001	<0·001
**Animal-based Pattern**						
**Female (n, %)**	5209(56·9)	5711(62·3)	5115(55·8)	3947(43·1)	<0·001	<0·001
**Age (Median,Q1, Q3,y)**	57·9(51·2;64·4)	59·2(51·8;65·8)	59·2(51·8;65·8)	58·1(51·1;64·0)	<0·001	0·608
**Age group (n, %)**					<0·001	0·911
**45-59 y**	5297(57·8)	4850(52·9)	4852(53·0)	5298(57·8)		
**60-74 y**	3544(38·7)	3761(41·0)	3768(41·1)	3529(38·5)		
**≥75 y**	321(3·5)	551(6·1)	542(5·9)	335(3·7)		

**Rural (n, %)**	6241(68·1)	5048(55·1)	5119(55·9)	5580(60·9)	<0·001	<0·001
**Income (n, %)**					<0·001	<0·001
**<5000 Yuan/month**	8039(87·7)	7166(78·2)	6848(74·7)	6928(75·6)		
**5000-9999 Yuan/month**	923(10·1)	1581(17·3)	1773(19·4)	1662(18·2)		
**≥10000 Yuan/month**	200(2·2)	415(4·5)	541(5·9)	572(6·2)		
**Han ethnicity (n, %)**	8181(89·3)	8440(92·1)	8420(91·9)	8250(90·0)	<0·001	0·13
**Educational level (n, %)**					<0·001	<0·001
**Below junior high school**	5476(59·8)	4945(54·0)	5215(56·9)	5161(56·3)		
**Junior high school**	3499(38·2)	3834(41·8)	3540(38·6)	3653(39·9)		
**Senior high school or above**	187(2·0)	383(4·2)	407(4·5)	348(3·8)		
**Having a partner (n, %)**	8653(94·4)	8559(93·4)	8655(94·5)	8752(95·5)	<0·001	<0·001
**Adequate physical activity (n, %)**	8039(87·7)	8040(87·8)	8239(89·9)	8425(92·0)	<0·001	<0·001
**Smoking (n, %)**	2224(24·3)	1994(21·8)	2359(25·7)	3134(34·2)	<0·001	<0·001
**Drinking (n, %)**	2681(29·3)	2992(32·7)	3392(37·0)	4066(44·4)	<0·001	<0·001
**BMI(Median, P25th, P75th,kg/m^2)**	24·4(22·1;26·9)	24·5(22·2;26·9)	23·9(21·7;26·3)	23·6(21·5;25·9)	<0·001	<0·001
**BMI group (n, %)**					<0·001	<0·001
**Underweight**	254(2·8)	258(2·8)	307(3·4)	345(3·8)		
**Normal weight**	3872(42·3)	3823(41·7)	4352(47·5)	4639(50·6)		
**Overweight**	3504(38·2)	3555(38·8)	3341(36·4)	3188(34·8)		
**Obesity**	1532(16·7)	1526(16·7)	1162(12·7)	990(10·8)		
**Family history of chronic diseases (n, %)**	4384(47·8)	4486(49·0)	4186(45·7)	3647(39·8)	<0·001	<0·001
**Diabetes Mellitus (n, %)**	904(9·9)	1183(12·9)	991(10·8)	905(9·9)	<0·001	0·156
**FPG(Median, P25th, P75th,mmol/L)**	5·2(4·8;5·7)	5·3(4·9;5·8)	5·3(4·9;5·8)	5·3(4·9;5·8)	<0·001	<0·001
**HbA1c (mean, std)**	5·1(0·9)	5·2(1·0)	5·14(1·0)	5·1(0·9)	<0·001	0·706
**Plant-based Pattern**						
**Female (n, %)**	5350(58·4)	5312(58·0)	4884(53·3)	4436(48·4)	<0·001	<0·001
**Age (Median,Q1, Q3,y)**	60·6(53·0;66·6)	59·3(51·9;65·6)	58·0(51·2;64·4)	56·1(50·4;63·0)	<0·001	<0·001
**Age group (n, %)**					<0·001	<0·001
**45-59 y**	4354(47·5)	4838(52·8)	5283(57·7)	5822(63·5)		
**60-74 y**	4224(46·1)	3815(41·6)	3531(38·5)	3032(33·1)		
**≥75 y**	584(6·4)	509(5·6)	348(3·8)	308(3·4)		
**Rural (n, %)**	6485(70·8)	5570(60·8)	5167(56·4)	4766(52·0)	<0·001	<0·001
**Income (n, %)**					<0·001	<0·001
**<5000 Yuan/month**	7898(86·2)	7434(81·1)	7085(77·4)	6564(71·6)		
**5000-9999 Yuan/month**	989(10·8)	1345(14·7)	1635(17·8)	1970(21·5)		
**≥10000 Yuan/month**	275(3·0)	383(4·2)	442(4·8)	628(6·9)		
**Han ethnicity (n, %)**	8327(90·9)	8433(92·0)	8428(92·0)	8103(88·4)	<0·001	<0·001
**Educational level (n, %)**					<0·001	<0·001
**Below junior high school**	6531(71·3)	5525(60·3)	4782(52·2)	3959(43·2)		
**Junior high school**	2523(27·5)	3410(37·2)	4013(43·8)	4580(50·0)		
**Senior high school or above**	108(1·2)	227(2·5)	367(4·0)	623(6·8)		
**Having a partner (n, %)**	8549(93·3)	8612(94·0)	8703(95·0)	8755(95·6)	<0·001	<0·001

**Adequate physical activity (n, %)**	8362(91·3)	8122(88·6)	8149(88·9)	8110(88·5)	<0·001	<0·001
**Smoking (n, %)**	2241(24·5)	2276(24·8)	2510(27·4)	2684(29·3)	<0·001	<0·001
**Drinking (n, %)**	2525(27·6)	2934(32·0)	3501(38·2)	4171(45·5)	<0·001	<0·001
**BMI(Median, P25th, P75th,kg/m^2)**	23·5(21·3;25·9)	24·0(21·8;26·4)	24·3(22·1;26·6)	24·6(22·4;27·0)	<0·001	<0·001
**BMI group (n, %)**					<0·001	<0·001
**Underweight**	403(4·4)	313(3·4)	247(2·7)	201(2·2)		
**Normal weight**	4698(51·3)	4249(46·4)	3973(43·4)	3766(41·1)		
**Overweight**	3047(33·3)	3330(36·3)	3582(39·1)	3629(39·6)		
**Obesity**	1014(11·0)	1270(13·9)	1360(14·8)	1566(17·1)		
**Family history of chronic diseases (n, %)**	3324(36·3)	3928(42·9)	4488(49·0)	4963(54·2)	<0·001	<0·001
**Diabetes Mellitus (n, %)**	984(10·7)	1079(11·8)	948(10·3)	972(10·6)	0·011	0·21
**FPG(Median, P25th, P75th,mmol/L)**	5·4(4·9;5·8)	5·3(4·9;5·8)	5·3(4·9;5·8)	5·2(4·9;5·7)	<0·001	<0·001
**HbA1c (mean, std)**	5·1(1·0)	5·2(1·0)	5·1(1·0)	5·1(1·0)	0·161	0·165

Table S3 The food and nutrient intake of the highest-scoring group (Q4) in the three dietary patterns

	Plant-based Pattern	Oriental traditional Pattern	Animal-based Pattern
Meat products and offal[d](g)	2·6	3·3	5·8
Dry legume[d](g)	8·5	3·1	3·6
Poultry[d](g)	9·8	15·2	13·7
Beverages[d](g)	11	13·8	28·7
Snacks[bc](g)	11·2	9·76	22·2
Other red meats[d](g)	11·7	6·5	23
Nuts[d](g)	14·1	8·3	24
Aquatic products[d](g)	27·4	49·4	31·4
Fungi and algae[d](g)	27·9	16·5	20·9
Legume products[d](g)	28·5	15·7	17·4
Liquors[ac](g)	43·4	76·6	76·8
Pork[d](g)	46·1	99·5	58·4
Other cereals[d](g)	50·2	7·53	19·4
Eggs[d](g)	53·6	33	44·4
Tubers[d](g)	80	26·4	65
Milk and dairy products[d](g)	93·2	40·5	113
Rice and products[d](g)	130	378	142
Fruits[ab](g)	166	97	166
Wheat and products[d](g)	182	38·9	151
Vegetables[d](g)	416	446	304
Vitamin b6[ab](mg)	0·04	0·03	0·04
Vitamin b12[ac](ug)	0·04	0·09	0·06
Riboflavin[d](mg)	1·01	1·12	1·04
Thiamin[d](mg)	1·14	1·12	1·14
Cu[ab](mg)	2·38	2·3	2·46
I[d](mg)	6·03	6·27	5·36
Mn[bc](mg)	6·84	7·35	6·72
Zn[d](mg)	12·6	15·1	13·4
Fiber[d](g)	15·9	13·5	14·8
Niacin[d](mg)	17·4	23·5	19·2
Fe(mg)	28·3	31·9	27·9
Vitamin e[ab](mg)	46·9	39·4	49·2
Se[bc](ug)	59·7	64·8	63
Protein[d](g)	81·3	91	84·7
Fat[d](g)	91·2	114	99·7
PUFA[d](g)	110	122	112
Vitamin c[d](mg)	118	128	112
MUFA[d](g)	142	190	156
SFA[d](g)	182	198	195
Cholesterol[d](mg)	192	304	231
Retinol[c](ug)	200	218	236

Folate[d](ug)	270	269	254
Carbohydrate[d](mg)	335	321	332
Mg[d](mg)	383	404	367
Ca[d](mg)	516	552	501
Vitamin a[ab](ug)	580	741	611
P(mg)	1235	1306	1240
K[a](mg)	2255	2321	2240
Carotene[d](ug)	2280	3142	2249
Na(mg)	2381	4214	2338
Energy[d](Kcal)	2448	2676	2541

a: There are significant differences between the Plant-based Pattern and the Oriental traditional Pattern·

b: There are significant differences between the Oriental traditional Pattern and the Animal-based Pattern·

c: There are significant differences between the Plant-based Pattern and the Animal-based Pattern·

d: There are significant differences among the three dietary patterns·

Table S4 The correlation between the three dietary patterns and food as well as nutrient intake

	Plant-based Pattern	Oriental traditional Pattern	Animal-based Pattern
Rice And Products	-0·26	0·77	-0·30
Wheat And Products	0·49	-0·53	0·33
Other Cereals	0·54	-0·34	0·13
Fungi And Algae	0·53	0·19	0·34
Tubers	0·42	-0·25	0·25
Dry Legume	0·39	0·05	0·09
Legume Products	0·57	0·12	0·18
Vegetables	0·35	0·44	-0·08
Fruits	0·47	0·06	0·45
Nuts	0·21	0·02	0·43
Pork	0·03	0·56	0·12
Other Red Meats	0·24	0·05	0·45
Meat Products And Offal	0·04	0·19	0·33
Poultry	0·13	0·37	0·30
Aquatic Products	0·18	0·54	0·22
Eggs	0·52	0·08	0·32
Milk And Dairy Products	0·28	-0·03	0·33
Liquors	0·09	0·17	0·18
Beverages	-0·01	0·14	0·26
Snacks	0·12	0·13	0·41
MUFA	-0·04	0·32	0·04
PUFA	0·04	0·12	0·03
SFA	0·06	0·17	0·12
Ca	0·15	0·21	0·06
Carbohydrate	0·15	-0·08	0·07
Cholesterol	-0·09	0·44	0·08
Cu	0·16	0·05	0·13
Diet Fiber	0·26	-0·07	0·13
Energy	0·07	0·14	0·08
Fat	-0·04	0·27	0·05
Fe	0·13	0·11	0·10
Folate	0·17	0·16	0·04
I	0·00	0·12	0·02
K	0·19	0·13	0·15
Mg	0·22	0·01	0·11
Mn	0·07	0·07	0·03
Na	0·10	0·12	0·11
Niacin	-0·06	0·40	0·04
P	0·17	0·13	0·14
Protein	0·12	0·21	0·15
Retinol	0·13	0·21	0·18

Riboflavin	0·13	0·25	0·14
Se	0·17	0·10	0·22
Thiamin	0·17	0·01	0·13
Carotene	-0·02	0·29	-0·07
Vitamin A	0·02	0·31	0·00
Vitamin B6	0·10	0·03	0·10
Vitamin B12	0·01	0·07	0·04
Vitamin C	0·07	0·19	-0·03
Vitamin E	0·13	-0·11	0·10
Zn	0·03	0·28	0·07

Table S5 Subgroup Analysis of the Association Between Dietary Patterns and T2DM in Different Populations

Oriental traditional pattern			Animal-based pattern			Plant-based pattern		
Subgroup	OR(95%CI)	Interaction Estimate<br>P for Interaction	Subgroup	OR(95%CI)	Interaction Estimate<br>P for Interaction	Subgroup	OR(95%CI)	Interaction Estimate<br>P for Interaction
**Male**								
Q1	1	0.95	Q1	1	0.93	Q1	1	0.90
Q2	1.07(0.91,1.25)	0.12	Q2	1.22(1.04,1.42)	0.02	Q2	1.07(0.92,1.25)	<0.01
Q3	1.03(0.88,1.21)		Q3	1.17(1.01,1.37)		Q3	0.96(0.83,1.13)	
Q4	1.09(0.93,1.27)		Q4	1.27(1.09,1.47)		Q4	0.88(0.76,1.03)	
**Female**								
Q1	1		Q1	1		Q1	1	
Q2	0.94(0.83,1.08)		Q2	1.25(1.11,1.41)		Q2	0.96(0.85,1.08)	
Q3	1.13(0.99,1.29)		Q3	1.08(0.95,1.22)		Q3	0.74(0.65,0.84)	
Q4	1.00(0.87,1.14)		Q4	1.04(0.90,1.20)		Q4	0.79(0.69,0.90)	
**Urban**								
Q1	1	0.93	Q1	1	0.50	Q1	1	0.96
Q2	1.10(0.94,1.30)	0.01	Q2	1.20(1.04,1.38)	1.02	Q2	1.04(0.90,1.20)	0.18
Q3	1.28(1.10,1.50)		Q3	1.08(0.94,1.25)		Q3	0.92(0.79,1.06)	
Q4	1.17(1.00,1.37)		Q4	1.11(0.96,1.29)		Q4	0.86(0.74,1.00)	
**Rural**								
Q1	1		Q1	1		Q1	1	
Q2	0.94(0.82,1.07)		Q2	1.27(1.11,1.44)		Q2	0.97(0.86,1.10)	
Q3	0.95(0.83,1.09)		Q3	1.13(0.98,1.30)		Q3	0.73(0.64,0.84)	
Q4	0.93(0.81,1.07)		Q4	1.17(1.02,1.35)		Q4	0.78(0.67,0.90)	
**Family history of chronic diseases(No)**								

Q1	1	0.96	Q1	1	1.02	Q1	1	0.97
Q2	1.00(0.87,1.15)	0.15	Q2	1.23(1.07,1.42)	0.50	Q2	1.04(0.91,1.19)	0.27
Q3	1.16(1.01,1.34)		Q3	1.04(0.89,1.20)		Q3	0.90(0.78,1.04)	
Q4	1.07(0.92,1.24)		Q4	1.15(0.99,1.33)		Q4	0.83(0.71,0.96)	
**Family history of chronic diseases(Yes)**								
Q1	1		Q1	1		Q1	1	
Q2	0.97(0.83,1.12)		Q2	1.24(1.09,1.41)		Q2	0.94(0.82,1.08)	
Q3	1.00(0.86,1.15)		Q3	1.18(1.03,1.34)		Q3	0.75(0.66,0.87)	
Q4	0.97(0.84,1.12)		Q4	1.13(0.98,1.30)		Q4	0.79(0.69,0.91)	
**BMI(<18·5 kg/m^2)**								
Q1	1	0.98	Q1	1	1.02	Q1	1	1.02
Q2	1.09(0.51,2.34)	0.35	Q2	2.04(0.74,5.65)	0.43	Q2	1.34(0.66,2.71)	0.31
Q3	1.18(0.50,2.79)		Q3	1.38(0.48,3.93)		Q3	0.61(0.24,1.53)	
Q4	1.14(0.43,3.01)		Q4	2.13(0.81,5.59)		Q4	0.37(0.12,1.21)	
**BMI(18·5-23·9 kg/ m^2)**								
Q1	1		Q1	1		Q1	1	
Q2	1.08(0.92,1.28)		Q2	1.20(1.01,1.43)		Q2	1.12(0.96,1.31)	
Q3	1.08(0.91,1.29)		Q3	1.02(0.86,1.22)		Q3	0.78(0.65,0.93)	
Q4	1.13(0.94,1.35)		Q4	1.15(0.96,1.37)		Q4	0.82(0.68,0.98)	
**BMI(24·0- 27·9 kg/ m^2)**								
Q1	1		Q1	1(1,1)		Q1	1	
Q2	0.88(0.75,1.03)		Q2	1.26(1.09,1.45)		Q2	0.93(0.80,1.08)	
Q3	1.01(0.86,1.17)		Q3	1.15(0.99,1.34)		Q3	0.83(0.72,0.96)	
Q4	0.92(0.79,1.07)		Q4	1.10(0.94,1.28)		Q4	0.79(0.67,0.92)	
**BMI(≥ 28·0 kg/m^2)**								
Q1	1		Q1	1		Q1	1	

Q2	1.04(0.83,1.31)		Q2	1.22(1.02,1.47)		Q2	0.92(0.75,1.13)	
Q3	1.15(0.93,1.44)		Q3	1.15(0.94,1.41)		Q3	0.85(0.69,1.04)	
Q4	1.08(0.87,1.34)		Q4	1.20(0.97,1.49)		Q4	0.86(0.70,1.06)	
**Drink(No)**								
Q1	1	1.02	Q1	1	1.03	Q1	1	1.02
Q2	0.96(0.85,1.08)	0.60	Q2	1.24(1.11,1.39)	0.44	Q2	0.99(0.89,1.10)	0.60
Q3	1.03(0.91,1.16)		Q3	1.07(0.95,1.21)		Q3	0.80(0.71,0.90)	
Q4	1.02(0.90,1.15)		Q4	1.11(0.98,1.26)		Q4	0.78(0.69,0.89)	
**Drink(Yes)**								
Q1	1		Q1	1		Q1	1	
Q2	1.09(0.90,1.32)		Q2	1.21(1.01,1.45)		Q2	1.02(0.84,1.23)	
Q3	1.22(1.01,1.46)		Q3	1.17(0.98,1.41)		Q3	0.89(0.74,1.08)	
Q4	1.07(0.89,1.29)		Q4	1.20(1.00,1.43)		Q4	0.90(0.75,1.08)	
**Smoke(No)**								
Q1	1	1.04	Q1	1	1.00	Q1	1	1.08
Q2	0.98(0.88,1.10)	0.27	Q2	1.27(1.15,1.42)	0.98	Q2	1.00(0.90,1.11)	0.04
Q3	1.10(0.98,1.23)		Q3	1.15(1.03,1.28)		Q3	0.8(0.71,0.89)	
Q4	1.02(0.91,1.14)		Q4	1.13(1.01,1.28)		Q4	0.81(0.72,0.9)	
**Smoke(Yes)**								
Q1	1		Q1	1		Q1	1	
Q2	1.01(0.81,1.25)		Q2	1.08(0.87,1.34)		Q2	0.99(0.80,1.23)	
Q3	1.03(0.83,1.26)		Q3	0.99(0.80,1.22)		Q3	0.92(0.75,1.14)	
Q4	1.07(0.86,1.32)		Q4	1.15(0.94,1.41)		Q4	0.87(0.70,1.07)	
