# Supplementary figures and images for: Geographical Distribution of Dietary Patterns and Their Association with T2DM in Chinese Adults Aged 45 y and Above: A Nationwide Cross-Sectional Study
Source: Nutrients. 2023 Dec 28;16(1):107. doi: 10.3390/nu16010107 (PMC10780680; doi:10.3390/nu16010107)

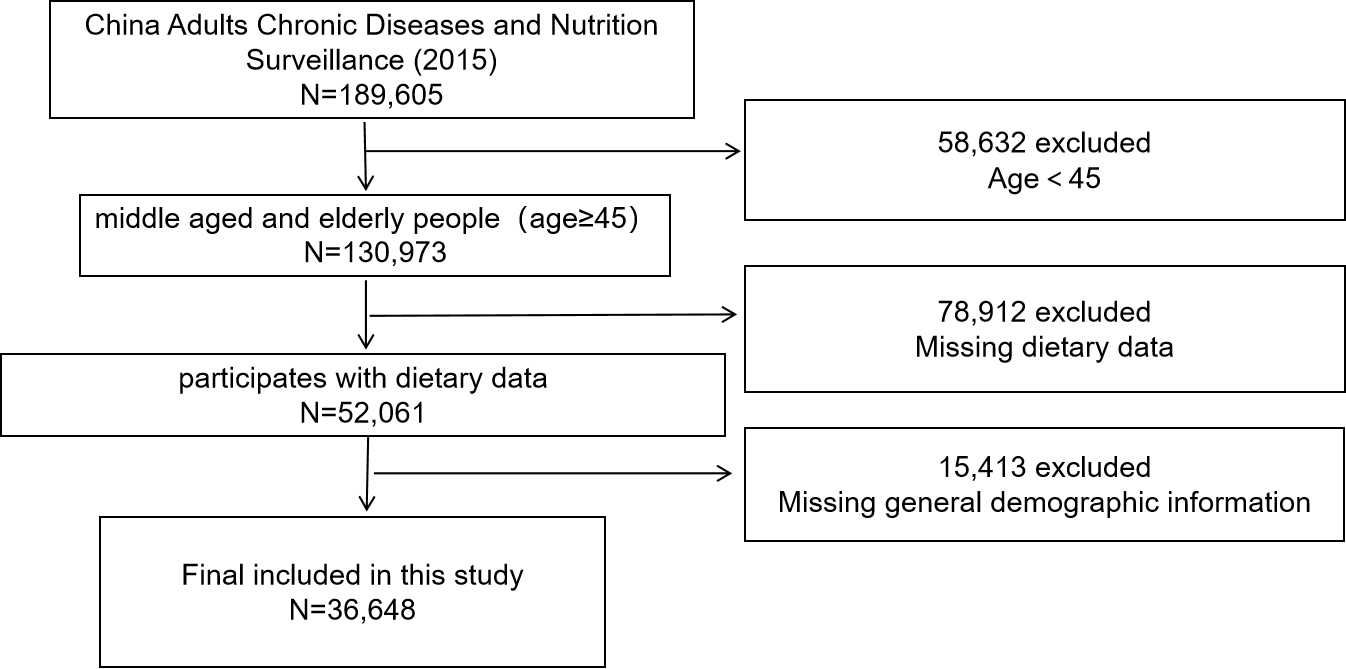

Supplement: Supplementary file 1 [file nutrients-16-00107-s001.zip › Figure S1.bmp]

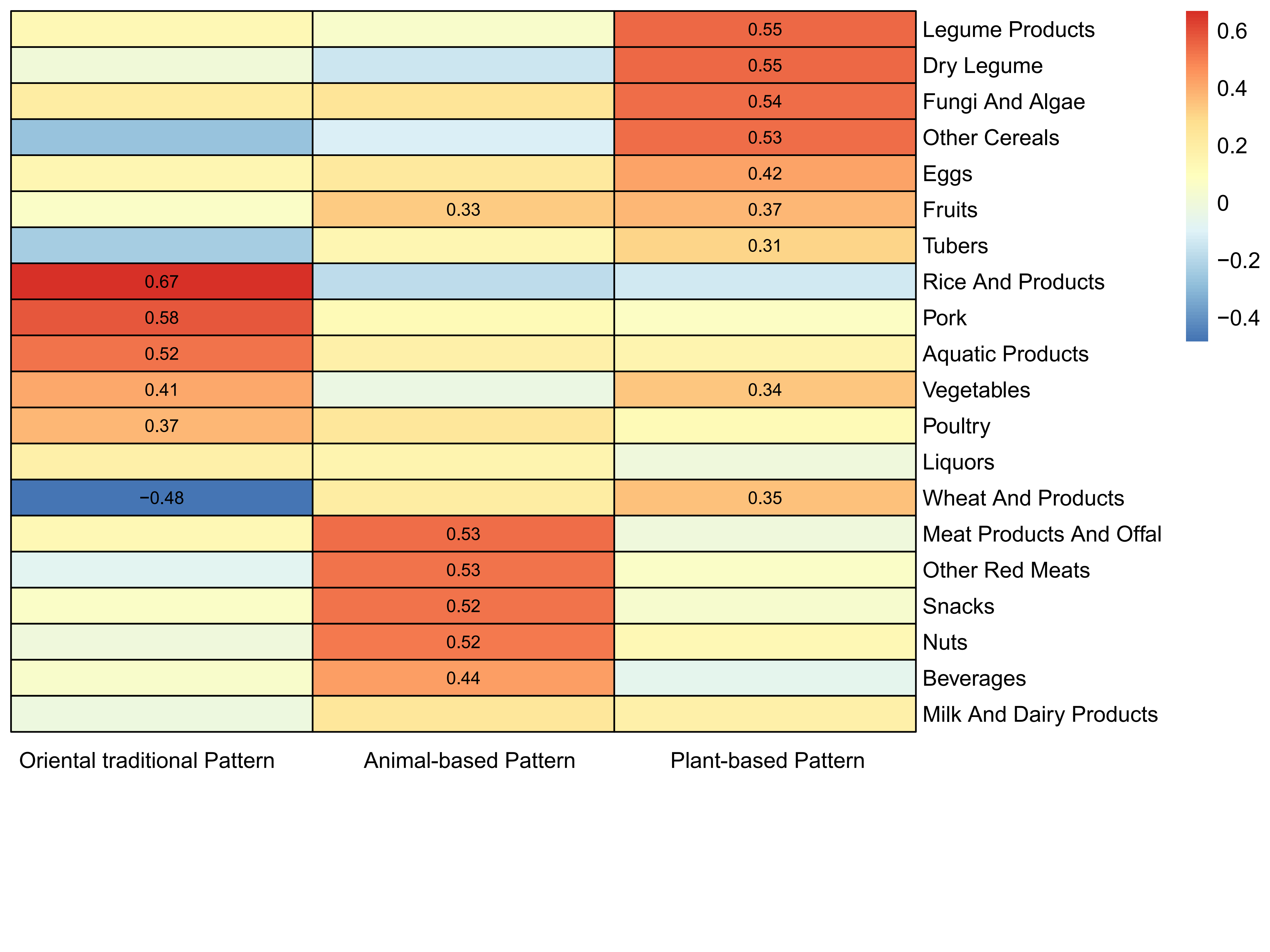

Supplement: Supplementary file 1 [file nutrients-16-00107-s001.zip › Figure S2.bmp]
